# Search engine optimization and its association with readability and accessibility of diabetic retinopathy websites

**DOI:** 10.1007/s00417-024-06472-3

**Published:** 2024-04-19

**Authors:** Matthew R. Lam, Garrett N. Manion, Benjamin K. Young

**Affiliations:** 1 Creighton University School of Medicine-Phoenix Regional Campus, Phoenix, AZ USA; 2 Creighton University School of Medicine, Omaha, NE USA; 3 Department of Ophthalmology, Casey Eye Institute, Oregon Health & Science University, Portland, OR USA

**Keywords:** Diabetic retinopathy, Readability, Search engine optimization, Patient education, Accessibility, Internet

## Abstract

**Purpose:**

This study investigated whether websites regarding diabetic retinopathy are readable for patients, and adequately designed to be found by search engines.

**Methods:**

The term “diabetic retinopathy” was queried in the Google search engine. Patient-oriented websites from the first 10 pages were categorized by search result page number and website organization type. Metrics of search engine optimization (SEO) and readability were then calculated.

**Results:**

Among the 71 sites meeting inclusion criteria, informational and organizational sites were best optimized for search engines, and informational sites were the most visited. Better optimization as measured by authority score was correlated with lower Flesch Kincaid Grade Level (*r* = 0.267, *P* = 0.024). There was a significant increase in Flesch Kincaid Grade Level with successive search result pages (*r* = 0.275, *P* = 0.020). Only 2 sites met the 6th grade reading level AMA recommendation by Flesch Kincaid Grade Level; the average reading level was 10.5. There was no significant difference in readability between website categories.

**Conclusion:**

While the readability of diabetic retinopathy patient information was poor, better readability was correlated to better SEO metrics. While we cannot assess causality, we recommend websites improve their readability, which may increase uptake of their resources.

**Supplementary Information:**

The online version contains supplementary material available at 10.1007/s00417-024-06472-3.


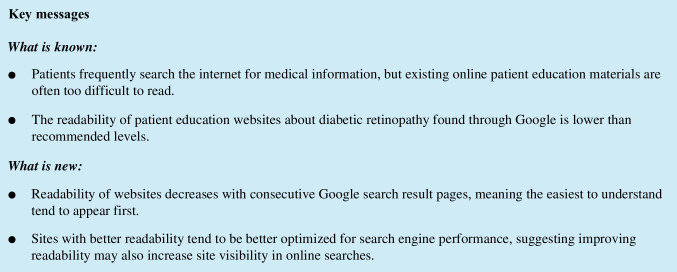


## Introduction

Diabetic retinopathy (DR) is the leading cause of blindness in working-age adults in the USA [1]. However, the risk of vision loss from DR can be reduced through early detection and management of blood sugar and blood pressure levels, as well as effective education [2]. In the current internet-centered information landscape, patients often turn to online search engines such as Google to access a wide range of materials related to their condition, with nearly 75% of patients using the internet as their first resource [3]. Some of the measurable factors that influence the effectiveness of search engine-driven patient education include the following: (1) search engine optimization (SEO), the process of designing a website such that it appears among the top search results, thereby impacting discoverability for patients, and (2) readability. The American Medical Association (AMA) recommends patient education material be at or below a 6th grade reading level as 54% of US adults read below this level [4, 5]. Patients with poor health literacy are more likely to have a lower understanding of their disease and will less often adhere to prescribed treatment plans [6, 7]. Inadequate health literacy is also associated with increased mortality and hospitalizations [8, 9]. In ophthalmology, limited health literacy is even associated with greater prevalence of DR [10]. Therefore, accessible online information may be important to address disparities in outcomes. With these considerations, this study aimed to assess the relationship between SEO and readability of websites with patient educational materials for DR found through internet searches.

## Methods

The authors queried the term “diabetic retinopathy” in the Google search engine on December 1st, 2022. Anonymous browsing was enabled via the browser’s “incognito mode” to observe the results that would be obtained by a patient searching the term for the first time by blinding the search engine to the investigators’ previous search history and habits. A bootstrapping study was performed to verify that the top 20 search results between various regions within the USA were similar by using a virtual proxy network (VPN) (Mozilla, San Francisco, CA, USA) to emulate geographic internet protocol (IP) address-based factors on search engine characteristics. Because regional results were nearly identical, the authors analyzed the default search results in Omaha, NE, USA for this study.

The authors screened the results listed on the first 10 pages (100 results) and classified whether they primarily provided patient-oriented information regarding DR based on self-identification (e.g., located in “For Patients” sections) and examination of website contents. In case of disagreements between authors, the senior author, a board-certified ophthalmologist and retina surgeon, acted as adjudicator. Websites judged to be targeted toward clinicians were excluded. The number of the search result page on which each site appeared was recorded. The authors also assigned the website category using the following criteria; the senior author again resolved disagreements. Academic websites were defined as those published by universities and other institutions of higher education. Commercial websites were defined as those published by corporations or manufacturers whose primary purpose was judged to be to advertise or provide information regarding a product or service. Informational websites were defined as those published by non-society or governmental organizations whose primary purpose is the dissemination of information. Organization websites were defined as those published by professional societies and governmental health organizations. Private practice websites were defined as those published on clinics’ websites that were not deemed academic.

SEO metrics were calculated using SemRush (Semrush Holdings Inc, Boston, MA, USA). Authority score (AS), a composite measure of overall website construction and SEO performance, was calculated for each website. AS is scored 0–100 and takes into account three elements: quantity and quality of backlinks (links from external websites), organic search traffic, and spam factors (indicators of artificial or illegitimate traffic). We selected AS due to its comprehensive approach, which encompasses backlink data, organic search data, and website traffic, offering a broad perspective on website construction and influence. Many other measurements exist, such as PageRank’s focus on backlink quantity and quality, Domain Authority’s backlink-based machine learning algorithm, and Trust Flow’s assessment of link trustworthiness [11]. Each of these metrics, while valuable, concentrates on more specific aspects of website evaluation, making AS a more holistic choice for our study’s objectives. SemRush also recorded website traffic for a period of November 1st to December 1st, 2022.

Readability for each website was quantified by Flesch Kincaid Grade Level via ReadablePro (Added Bytes Ltd, Horsham, UK) [12]. Flesch Kincaid Grade Level is the most commonly used readability tool to estimate reading grade level and has become a cornerstone in the examination of patient-oriented materials [13–19]. It calculates the estimated grade level required to read a document using two criteria, words per sentence and syllables per word. The calculated grade levels were evaluated in reference to the 6th grade reading level recommended for patient materials by the AMA, and the 8th grade level previously recommended by the National Institutes of Health (NIH) [5, 20, 21].

The authors conducted statistical analysis in SPSS version 28.0.0.0 (IBM, Armonk, NY, USA). One-way ANOVA and post-hoc Tukey test were performed to identify differences in website readability, AS, and monthly visits between categories. Linear regression was performed to determine a trend in readability with successive search result pages and to evaluate relationships between readability and AS and monthly visits. Finally, linear regression was also performed with AS and monthly visits to confirm correlation as measures of SEO. Significance was defined as *P* < 0.05.

## Results

Seventy-one websites met the inclusion criteria; their search result position and categories are detailed in Table 1. The authors judged 29 websites to be clinician-focused and excluded them from the analysis. Private practice websites were the most common, comprising 45% of included search results. Across all sites analyzed, the average Flesch Kincaid Grade Level was 10.5. Using the 6th grade reading level recommended for patient materials by the AMA, 2 (3%) of the 71 sites were appropriately readable. When instead considering the less stringent 8th grade level recommendation proposed by the NIH, 13 (18%) sites met readability recommendations.

**Table 1:** Distribution of websites by search result page and website category

Search result page	Sites, *N* (%)	Website category	Sites, *N* (%)
1	9 (12.7)	Private	32 (45.1)
2	10 (14.1)	Academic	15 (21.1)
3	7 (9.9)	Informational	12 (16.9)
4	9 (12.7)	Organization	9 (12.7)
5	10 (14.1)	Commercial	3 (4.2)
6	6 (8.5)		
7	4 (5.6)		
8	3 (4.2)		
9	6 (8.5)		
10	7 (9.9)		
Total	71 (100)		

Website SEO and readability metrics by category are detailed in Table 2. The organization category had the highest AS at 49, significantly greater than all other categories except informational, which had the second highest AS at 31; the AS of the organization category was 45 higher than that of the private practice category (*P* < 0.001), 42 higher than that of the commercial category (*P* = 0.008), and 24 higher than that of the academic category (*P* = 0.024). Private practice websites had the lowest AS of 4, significantly lower than organizational, informational (lower by 27, *P* < 0.001), and academic sites (lower by 21, *P* = 0.003). Additional detail regarding distribution of AS is available in Online Resource 1. AS and monthly visits correlated with each other well as measurements of SEO (*r* = 0.60, *P* < 0.001). However, informational sites had more monthly visits (5.3 × 10^7^ visits/month) than organizational sites (4.8 × 10^7^ visits/month) on average, and the only significant difference was between informational and private sites (8.7 × 10^5^ visits/month) (5.3 × 10^6^ more visits, *P* = 0.033). Academic sites had 2.0 × 10^7^ visits/month and commercial sites had the lowest at 1.1 × 10^4^ visits/month.

**Table 2:** Average SEO and readability metrics by website category

Website category	Average authority score	Average website visits	Average Flesch Kincaid Grade Level
Academic	25	19,905,636	9.6
Commercial	7	11,253	10.6
Informational	31	53,619,531	11.0
Organization	49	48,511,946	10.5
Private	4	874,377	10.7

The mean Flesch Kincaid Grade Level by website category showed no significant difference between categories (Online Resource 2). The mean grade level by category was academic at 9.6, commercial at 10.6, informational at 11.0, organization at 10.5, and private at 10.7. These were all above the NIH and AMA recommended grade levels. There was a significant positive association between Flesch Kincaid Grade Level and increasing search result page (*r* = 0.275, *P* = 0.020) (Online Resource 3). Comparing readability and SEO metrics, Flesch Kincaid Grade Level demonstrated a negative correlation with AS (*r* = −0.267, *P* = 0.024) (Online Resource 4) and website traffic volume (*r* =  −0.30, *P* = 0.012).

## Discussion

In this study, our key findings were (1) the vast majority (97%) of diabetic retinopathy educational websites exceed the median level of health literacy in the USA, (2) better readability as measured by Flesch Kincaid Grade Level was correlated with improved SEO, and (3) more readable pages tended to appear earlier in search results. Despite readability tending to improve with earlier search results, the first of two websites which met the target reading level appeared on the third page of search results. Readers are unlikely to access these readable websites, as over 90% of individuals using the Google search engine do not select websites shown past the first page of results [22]. The results of this study demonstrated that patient education websites for DR are currently written in language with poor readability and thus limit their effectiveness in patient education. Low readability may disproportionately affect already vulnerable groups and exacerbate demographic-based healthcare disparities as individuals of minority ethnic or lower socioeconomic backgrounds tend to have lower levels of health literacy [23], and diabetic retinopathy is more prevalent in minority ethnic groups compared to non-Hispanic whites, particularly among African Americans, Hispanics, and Native Americans [1, 24].

These findings were consistent with existing research into readability of materials for other ophthalmologic and general medical conditions that similarly found that reading levels routinely exceed recommendations [13, 15–19]. However, other studies used more curated methodology, such as a 2019 article by Kloosterboer et al., which reported that 11 handpicked, reputable, and professionally produced DR websites wrote at an average of an 11th grade reading level with varying levels of accuracy and completeness [25]. While this investigation demonstrated concerns with readability, as does the present study, the 2019 analysis did not emulate the organic search experience of patients. While the authors selected websites known to them to be reputable, patients may not be familiar with the various sources presented in search results and instead rely more on the order in which they are presented. Therefore, focusing the investigation on sites that are present at the top of search results, rather than strictly professional sources, would likely better represent the patient experience. The present study is the first to our knowledge to consider this limited patient understanding by analyzing DR website readability in the context of organic search results and subsequently complement these findings with novel insights into SEO, an important determinant of said search result order.

Greater readability as represented by lower Flesch Kincaid Grade Levels was associated with higher SEO metrics, suggesting that SEO and readability are related and that more readable sites more frequently appear among top search results. This was further evidenced by earlier search result pages having lower average reading levels than later pages. Therefore, improving readability may benefit patients by not only increasing understandability of a given website but also by increasing that website’s visibility and thus the probability that patients will access it. The mechanism through which readability and SEO are associated is unclear. Readability may directly contribute to SEO, or it may act as an indirect factor; for instance, greater engagement on a given site is known to increase its SEO, but less readable material may cause patients to exit a page quickly. As Flesch Kincaid Grade Level penalizes long sentences and complex words, focusing on shorter, more concise sentences and smaller, less technical words may be effective strategies for website publishers seeking to improve their content and potentially increase website traffic. However, despite the association, the design methodology could not establish a causal relationship.

While there were no significant differences in readability between website categories, informational and organizational sites were better optimized for search engines and more visited than other website types. Therefore, enhanced readability of websites in these widely accessed categories would benefit the most patients. Further, practice websites that seek to drive increased traffic may benefit by improving their readability.

Basic, fundamental steps to improve SEO that can be taken individually include ensuring detailed yet succinct HTML headers, such as <title> and meta description, are included for each page on the site, webpage sections are demarcated with heading tags, images have descriptive alt text, site navigation is intuitive (e.g., use of breadcrumb navigation), and that the use of keywords in website URLs, headings, and website content are frequent and tailored to the target audience. Other, more complex strategies that may require partnership with website development teams include better optimizing websites for mobile devices, reducing loading times, including structured data markup to allow search engines to feature snippets of site content among search results, and creating a backlink network, the details of which are beyond the scope of this discussion. Finally, tools like Google Analytics can help monitor the performance of one’s website and highlight points of potential improvement [26].

A recent development in online patient education involves patients using artificial intelligence (AI) and large language models (LLMs) like ChatGPT to seek out medical information. While AI has the potential to make complex information more accessible, its real-world efficacy varies. A study by Momenaei et al. (2023) revealed that ChatGPT-4 often generates responses at a high reading level, challenging for the average reader to comprehend [27]. Another major concern observed is the AI providing inaccurate or misleading information, known as “hallucinations” [28]. Though more specific prompting by patients can guide LLMs to create more accessible text, hallucinations can only be addressed by the LLM creators modifying the model itself. Given the unpredictable accuracy and readability in AI-generated medical content, improved accessibility of well-written patient materials has become even more crucial to avoid pushing patients away from traditional websites and toward AI.

There are several limitations of this observational study. Quantification of readability relied upon a previously established readability formula which considers sentence structure and syllabalism to estimate grade level and reading ease. Website layout and presence of images, videos, and advertisements may also affect readability. The categorization of websites performed in this study may also have been too rigid, as the modern approach of diverse organizations often blurs the lines between “private” and “academic,” which may affect the target audience, research goals, and more. Another important consideration to the applicability of this study includes the volatility of search engine results; our search query will not be identically reproducible due to the time and location of our search.

Overall, this study revealed that the majority of online DR patient material found through search engine query exceeded the recommended and average adult reading level in the USA and that improving readability may improve SEO. This obfuscation of accessible information reflects a disconnect in health literacy between the medical community and patients and should be rectified by developing more accessible informational resources or updating existing ones with special consideration to readability and SEO to maximize patient understanding and discoverability.

## Supplementary Information

Below is the link to the electronic supplementary material.

Supplementary file1 (PDF 58 KB)

Supplementary file2 (PDF 89 KB)

Supplementary file3 (PDF 96 KB)

Supplementary file4 (PDF 65 KB)
