# Supplementary material for: Search engine optimization and its association with readability and accessibility of diabetic retinopathy websites
Source: Graefes Arch Clin Exp Ophthalmol. 2024 Apr 19;262(9):3047–52. doi: 10.1007/s00417-024-06472-3 (PMC11377497; doi:10.1007/s00417-024-06472-3)
Supplement: Supplementary file 1 — Supplementary file1 (PDF 58 KB) [file 417_2024_6472_MOESM1_ESM.pdf]

Figure S1

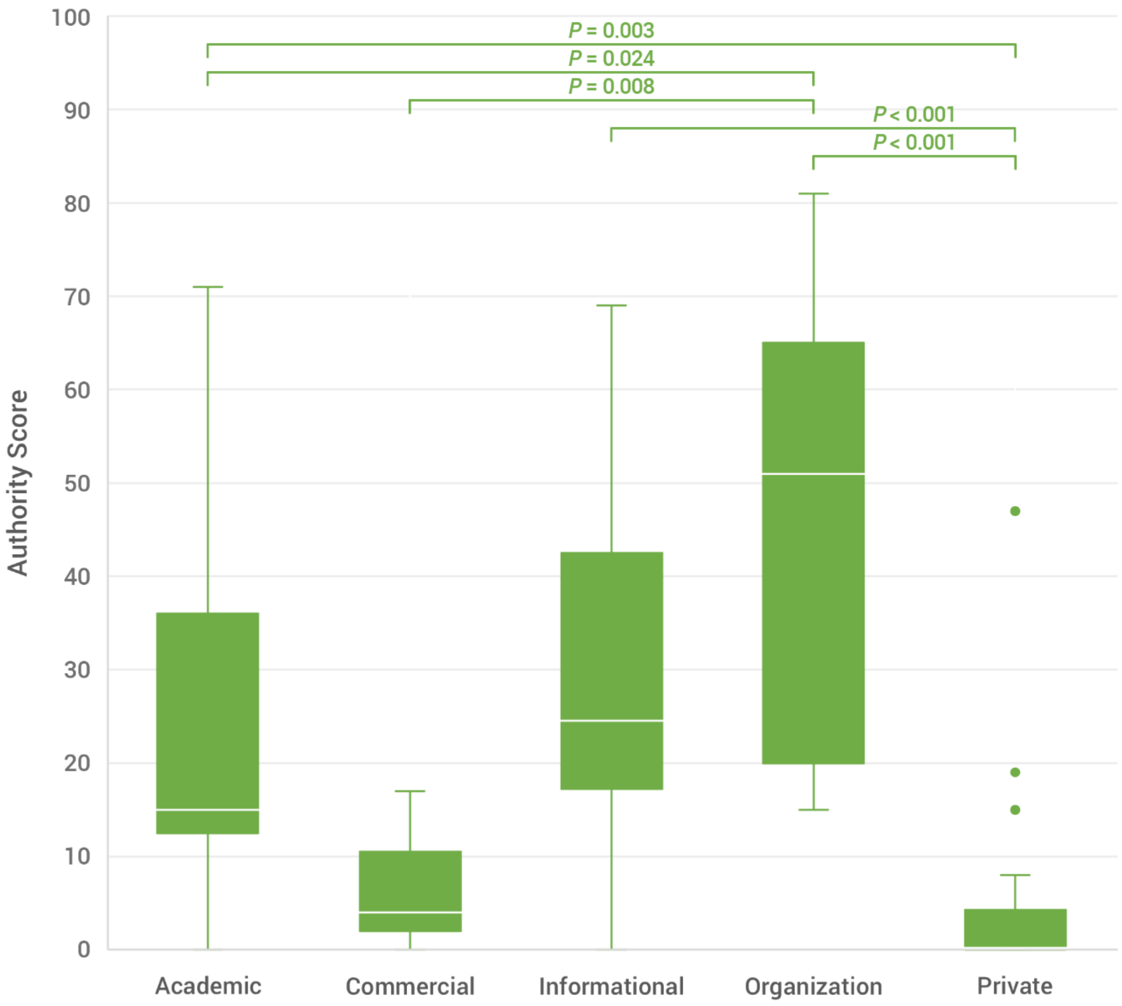

Distribution of Authority Scores of websites by their source categories. Significant differences in mean Authority Score between categories are indicated by brackets and corresponding P-values.
