# Supplementary material for: Search engine optimization and its association with readability and accessibility of diabetic retinopathy websites
Source: Graefes Arch Clin Exp Ophthalmol. 2024 Apr 19;262(9):3047–52. doi: 10.1007/s00417-024-06472-3 (PMC11377497; doi:10.1007/s00417-024-06472-3)
Supplement: Supplementary file 2 — Supplementary file2 (PDF 89 KB) [file 417_2024_6472_MOESM2_ESM.pdf]

Figure S2

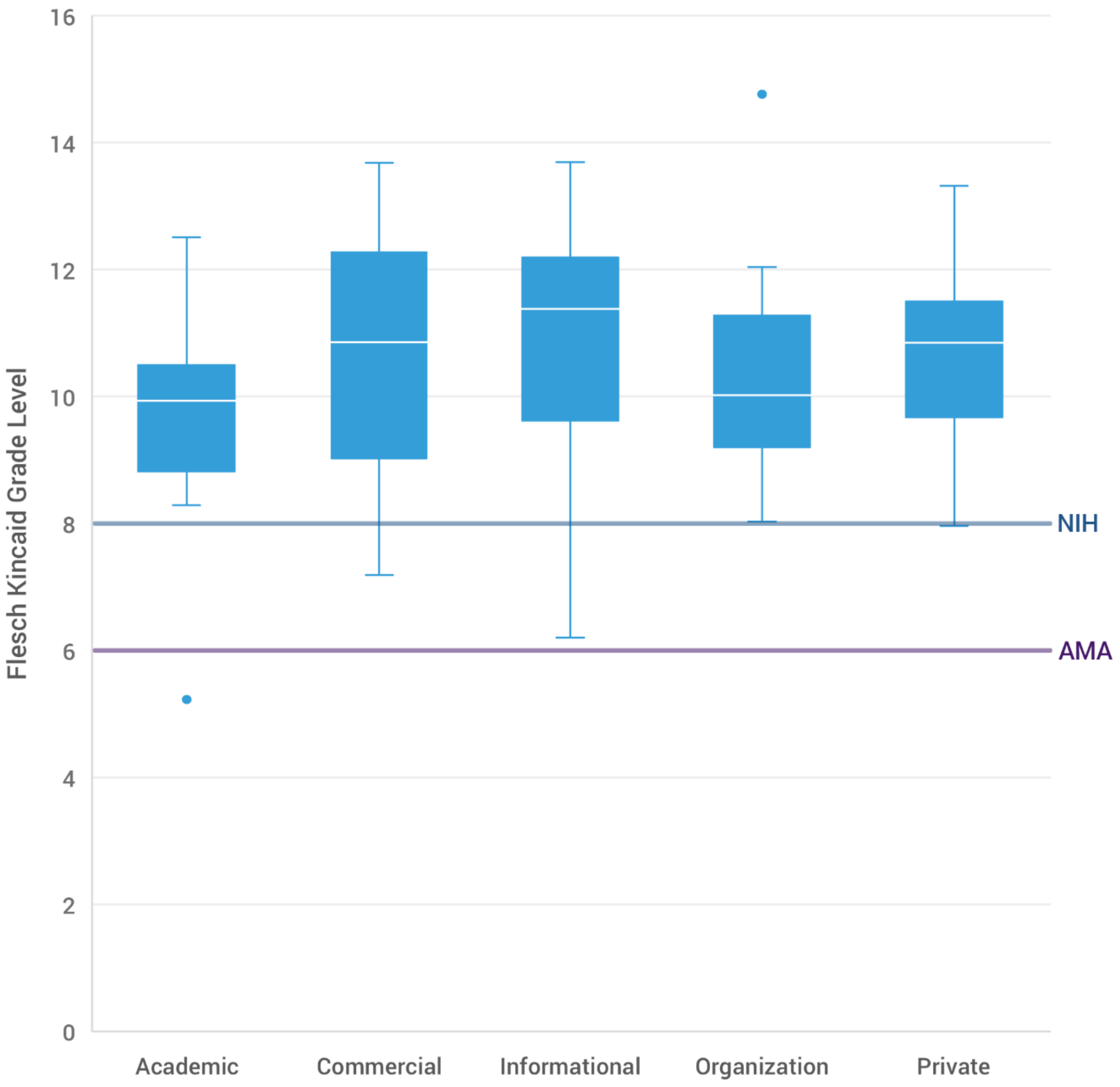

Distribution of Flesch Kincaid Grade Levels of websites by their source categories. The blue line labeled “NIH” denotes the 8th grade reading level recommended by the NIH. The purple line labeled “AMA” denotes the 6th grade reading level recommended by the AMA.
