# Supplementary material for: Search engine optimization and its association with readability and accessibility of diabetic retinopathy websites
Source: Graefes Arch Clin Exp Ophthalmol. 2024 Apr 19;262(9):3047–52. doi: 10.1007/s00417-024-06472-3 (PMC11377497; doi:10.1007/s00417-024-06472-3)
Supplement: Supplementary file 3 — Supplementary file3 (PDF 96 KB) [file 417_2024_6472_MOESM3_ESM.pdf]

Figure S3

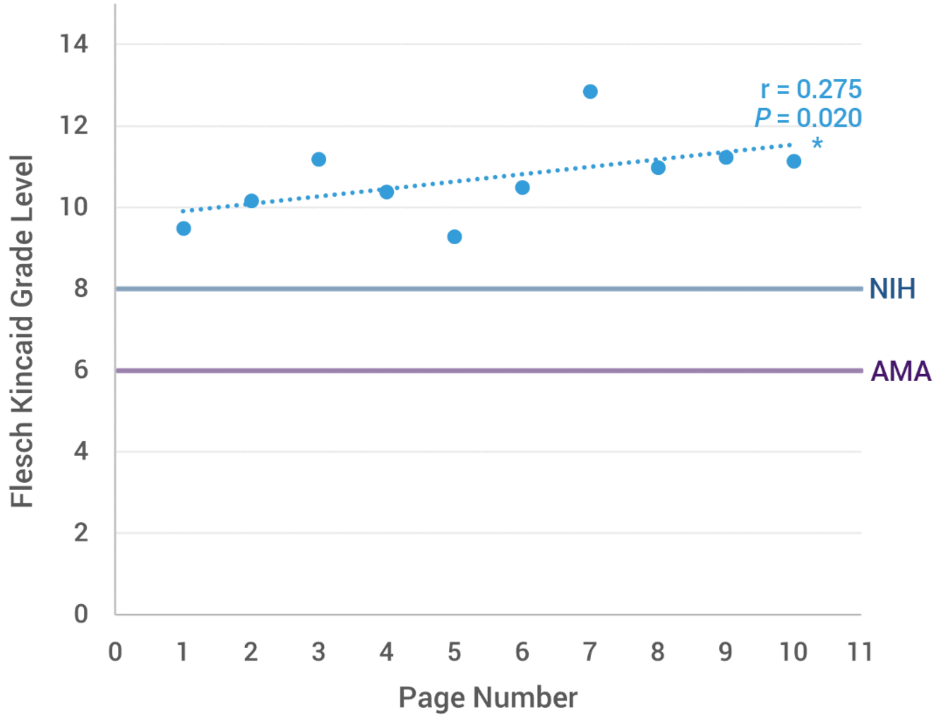

Mean Flesch Kincaid Grade Levels of websites by search result page. The dotted line represents the line of best fit; the asterisk and corresponding r- and P-values indicate the strength and significance of the correlation. The blue line labeled “NIH” denotes the 8th grade reading level recommended by the NIH. The purple line labeled “AMA” denotes the 6th grade reading level recommended by the AMA.
