# Supplementary material for: Search engine optimization and its association with readability and accessibility of diabetic retinopathy websites
Source: Graefes Arch Clin Exp Ophthalmol. 2024 Apr 19;262(9):3047–52. doi: 10.1007/s00417-024-06472-3 (PMC11377497; doi:10.1007/s00417-024-06472-3)
Supplement: Supplementary file 4 — Supplementary file4 (PDF 65 KB) [file 417_2024_6472_MOESM4_ESM.pdf]

Figure S4

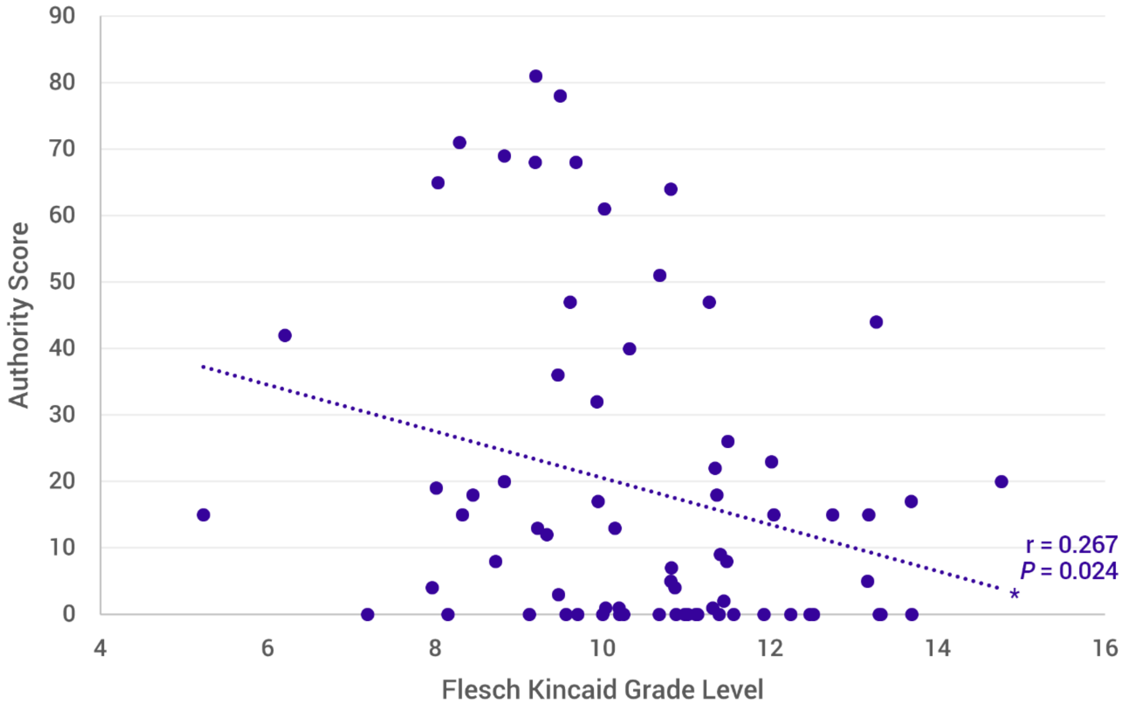

Correlation of the Authority Scores and Flesch Kincaid Grade Levels of websites. The dotted line represents the line of best fit; the asterisk and corresponding r- and P-values indicate the strength and significance of the correlation.
